# In situ analysis of FGFR2 mRNA and comparison with *FGFR2* gene copy number by dual-color in situ hybridization in a large cohort of gastric cancer patients

**DOI:** 10.1007/s10120-017-0758-x

**Published:** 2017-08-29

**Authors:** Yasutoshi Kuboki, Christoph A. Schatz, Karl Koechert, Sabine Schubert, Janine Feng, Sabine Wittemer-Rump, Karl Ziegelbauer, Thomas Krahn, Akiko Kawano Nagatsuma, Atsushi Ochiai

**Affiliations:** 1 National Cancer Center Hospital East Kashiwa, Kashiwa, Japan; 2 Bayer AG, Muellerstr. 178, 13353 Berlin, Germany; 3 Ventana Medical Systems Inc., Oro Valley, AZ USA; 4 National Cancer Center, Exploratory Oncology Research and Clinical Trial Center, Tokyo, Japan

**Keywords:** Fibroblast growth factor receptors 2, Stomach neoplasms, In situ hybridization, Gene amplification, Molecular targeted therapy

## Abstract

**Background:**

Fibroblast growth factor receptor (FGFR2) has been proposed as a target in gastric cancer. However, appropriate methods to select patients for anti-FGFR2 therapies have not yet been established.

**Methods:**

We used in situ techniques to investigate FGFR2 mRNA expression and gene amplification in a large cohort of 1036 Japanese gastric cancer patients. FGFR2 mRNA expression was determined by RNAscope. FGFR2 gene amplification was determined by dual-color in situ hybridization (DISH).

**Results:**

We successfully analyzed 578 and 718 samples by DISH and RNAscope, respectively; 2% (12/578) showed strong *FGFR2* gene amplification (FGFR2:CEN10 >10); moderate *FGFR2* gene amplification (FGFR2:CEN10 <10; ≥2) was detected in 8% (47/578); and high FGFR2 mRNA expression of score 4 (>10 dots/cell and >10% of positive cells with dot clusters under a 20× objective) was seen in 4% (29/718). For 468 samples, both mRNA and DISH data were available. FGFR2 mRNA expression levels were associated with gene amplification; FGFR2 mRNA levels were highest in the highly amplified samples (*n* = 12). All highly amplified samples showed very strong FGFR2 mRNA expression (dense clusters of the signal visible under a 1× objective). Patients with very strong FGFR2 mRNA expression showed more homogeneous FGFR2 mRNA expression compared to patients with lower FGFGR2 mRNA expression. Gastric cancer patients with tumors that had an FGFR2 mRNA expression score of 4 had shorter RFS compared with score 0–3 patients.

**Conclusion:**

RNAscope and DISH are suitable methods to evaluate FGFR2 status in gastric cancer. Formalin-fixed paraffin-embedded (FFPE) tissue slides allowed evaluation of the intratumor heterogeneity of these FGFR2 biomarkers.

**Electronic supplementary material:**

The online version of this article (doi:10.1007/s10120-017-0758-x) contains supplementary material, which is available to authorized users.

## Introduction

Gastric cancer is the third most frequent cause of death from cancer worldwide [1]. Worldwide, more than 700,000 patients per year die of gastric cancer [2, 3]. Surgery remains the only curative treatment option. Response to chemotherapy for inoperable, advanced-stage patients is limited, with median survival time less than 1 year [4]. More recently, targeted agents have shown a benefit in gastric cancer. The ToGA trial demonstrated a survival benefit of trastuzumab, an anti-human epidermal growth factor receptor 2 (HER2)-targeting antibody, in combination with chemotherapy for HER2-positive advanced gastric cancer patients [5]. HER2 amplifications or overexpression have been reported in 7–34% of the investigated tumors [6]. In 2014, the vascular endothelial growth factor receptor 2 (VEGFR2) antibody ramucirumab, which was approved for treatment of gastric cancer in an unselected patient population, was the second targeted agent [7]. Epidermal growth factor receptor (EGFR)-directed antibodies cetuximab or panitumumab have failed to provide a significant benefit in non-molecularly selected gastric cancer patients [8, 9]. The anti-EGFR antibody nimotuzumab is currently being investigated in phase 3 clinical trials in patients with EGFR-overexpressing advanced gastric cancer (AGC) (ENRICH study NCT01813253). Agents inhibiting hepatocyte growth factor receptor (c-MET) signaling, such as the anti-MET antibody onartuzumab or the small molecular inhibitor tivantinib, have been evaluated in gastric cancer without achieving sufficient clinical benefits [10, 11].

Overexpression and gene amplification of fibroblast growth factor receptors 2 (FGFR2) has been associated with poor outcome in gastric cancer [12, 13]. Different methodologies have been used to analyze FGFR2 expression and gene amplification in gastric cancer: Southern blot analysis [14], comparative genomic hybridization [15, 16], quantitative real-time polymerase chain reaction (qPCR) [17], and fluorescence in situ hybridization (FISH) [17]. Most selected biomarker clinical trials in gastric cancer have been designed on the basis of tissue-based methods such as immunohistochemistry (IHC) or in situ hybridization (ISH) [5, 9].

More recently, mRNA in situ hybridization, FISH, and immunohistochemistry (IHC) for the assessment of FGFR2 in gastric cancer have been directly compared [18]. Intratumoral heterogeneity is commonly observed in gastric cancer and has been observed for *FGFR2* gene copy number [19] and for FGFR2 mRNA and protein expression [18]. Treatment with a targeted agent is usually based on a diagnostic test demonstrating the presence of the target in a diagnostic biopsy. However, the target might not be present in all the cells of the primary lesion. The level of clonal heterogeneity may reduce the therapeutic effect of the drug on the tumor as a whole and lead to a poor response [20]. Evolutionary adaption and heterogeneous expression of a target protein have been discussed to limit the value of targeted therapies [21]. To further investigate FGFR2 as a target in gastric cancer, we were interested in the intra-tumor heterogeneity of the FGFR2 mRNA expression and gene amplification and the association with clinical outcome. We therefore used dual-color in situ hybridization (DISH) to detect *FGFR2* gene copy number and the mRNA in situ hybridization technique called RNAscope to detect FGFR2 mRNA expression levels. Use of light microscopy allowed the assessment of intratumor heterogeneity in a large set of Japanese gastric cancer patients. We also correlated *FGFR2* gene amplification and FGFR2 mRNA expression with patient outcome to further investigate the biological significance of FGFR2 in gastric cancer.

## Materials and methods

### Patients and data collection

Anonymized tissue samples from 1036 patients with gastric adenocarcinoma (GAC) who underwent curative resection of primary tumor and lymph node dissection (D1 or D2) at the National Cancer Center Hospital East (Kashiwa, Japan) between January 2003 and July 2007 were collected [22]. Among the patients, 119 patients received adjuvant or preoperative chemotherapies (mainly fluoropyrimidine monotherapy). Sample collection was performed in agreement with the principles of good clinical practice according to the Declaration of Helsinki (1964). Every patient signed an informed consent for sample collection. A pathological report and hematoxylin and eosin (H&E)-stained slides were reviewed for the tumor parameters, including histopathology, depth of tumor invasion and capillary invasion status, such as lymphatic and venous invasion, and lymph node metastasis. Staging and histopathology were conducted according to the Japanese classification of gastric carcinoma, third English edition [23]. The study protocol was approved by the institutional review board at the National Cancer Center, Japan (2013-157). Clinicopathological characteristics are shown in Supplementary Tables 1 and 2.

### Tissue microarray construction

Representative tumor areas were selected and marked on H&E-stained slides for the construction of tissue microarrays (TMAs). Two 2.0-mm-diameter tumor cores were obtained from the same tissue block in each case using a manual tissue arrayer (Azumaya Ika Kikai, Tokyo, Japan). These cores were assembled in a TMA. Each TMA block contained 48 cores.

### RNA in situ hybridization

FGFR2 mRNA expression was determined by RNAscope 2.0 following Advanced Cell Diagnostics (ACD, Newark, CA, USA) kit instructions. In brief, freshly cut 3-µm formalin-fixed paraffin-embedded (FFPE) slides were baked for 1 h at 60 °C. Samples were deparaffinized and pre-treated. Target probes for FGFR2 and peptidylprolylisomerase (PPIB) as control were hybridized. Signal was amplified and developed. Counterstaining with H&E was performed. Scoring was done according to the kit instruction: no staining or less than 1 dot/cell under a 40× objective lens (score 0); 1–3 dots/cell under a 20–40× objective lens (score 1); 4–10 dots/cell and no or very few clusters of dots under a 20–40× objective lens (score 2); >10 dots/cell and <10% of positive cells with dot clusters under a 20× objective lens (score 3), and >10 dots/cell and >10% of positive cells with dot clusters under a 20× objective (score 4). Samples with dense clusters of RNAscope signal visible under a 1× objective were in some analyses categorized as score 5. Samples with neither PPIB nor FGFR2 signal were excluded from the analysis.

### DISH

We used dual-color in situ hybridization (DISH) and analyzed the FGFR2 signals per tumor cell and a chromosome 10 probe as reference, as the *FGFR2* gene is localized on human chromosome 10. This method determines the *FGFR2* gene amplification and allows assessment of the location and distribution of areas of *FGFR2* gene amplification in the tumor at the same time. The FGFR2/chromosome 10 dual ISH (DISH) assay was carried out on a Ventana Benchmark XT staining platform. Signals from 50 nuclei representing the highest copy number for that specimen per slide were counted. Moderate *FGFR2* gene amplification was defined by a ratio of *FGFR2* gene signal to chromosome 10 signal (CEN10) of less than ten but more than two (FGFR2:CEN10 <10; ≥2). High *FGFR2* gene amplification was defined by a ratio of FGFR2:CEN10 larger than ten.

### Statistical analysis

All analyses were done in R v3.2.3 [R Core Team (2015). R: a language and environment for statistical computing. R Foundation for Statistical Computing, Vienna, Austria. URL https://www.R-project.org/]. For analysis of both overall survival (OS) and recurrence-free survival (RFS), univariate Cox proportional hazards models and Kaplan–Meier estimates were computed, complemented by a multivariate Cox model analysis adjusting for clinical covariates. The latter models were moreover subjected to Akaike information criterion (AIC)-based backward variable selection to ensure robust and informative statistical modeling.

## Results

**Fig. 1:**
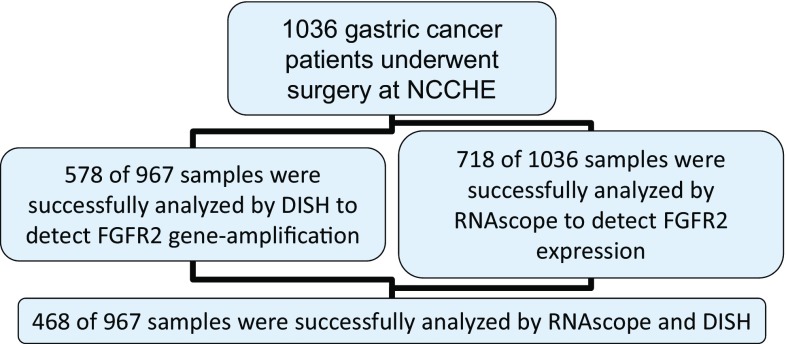
Patient samples flow diagram. *DISH* dual-color in situ hybridization, *FGFR* fibroblast growth factor receptor

**Fig. 2:**
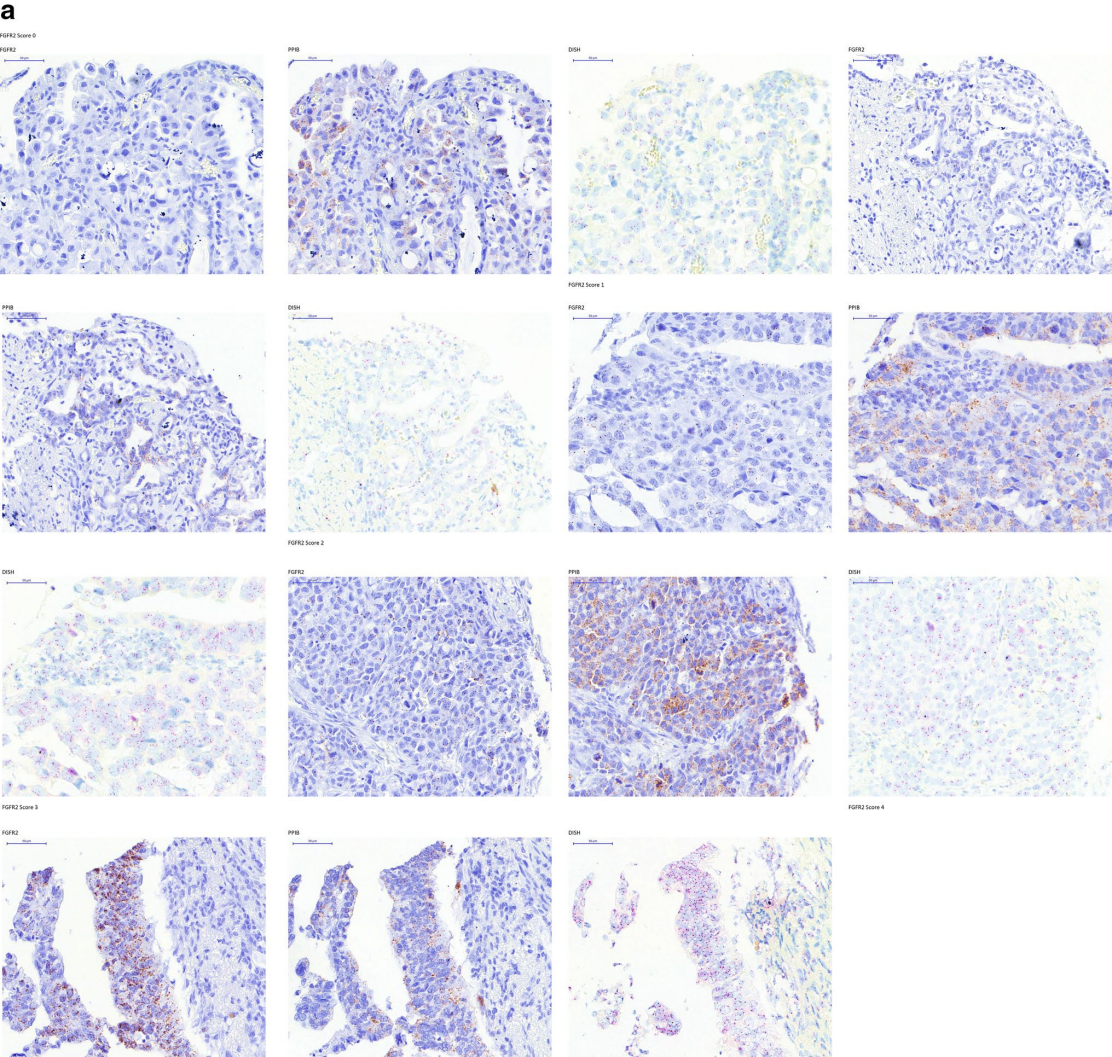

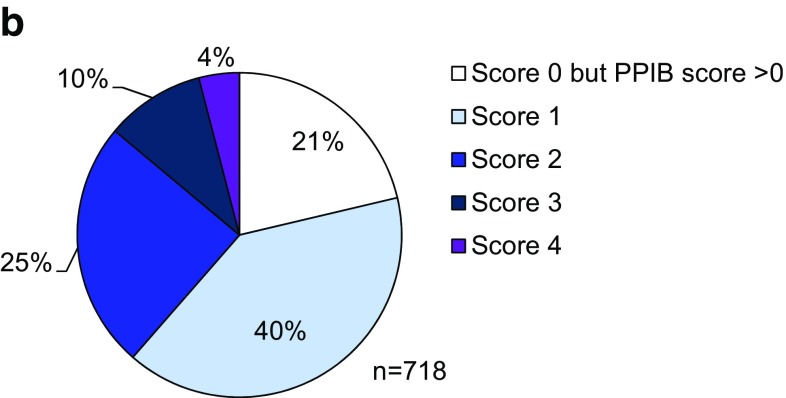
Fibroblast growth factor receptor (FGFR)2 mRNA analysis. FGFR2 mRNA expression was determined by RNAscope 2.0 following manufacturer’s instructions (Advanced Cell Diagnostics, Hayward, CA, USA) using FGFR2 and peptidyl prolyl isomerase B (PPIB) target probes. FGFR2 expression was scored on a scale from 0 to 4. **a** Representative images. **b** Prevalence of FGFR2 mRNA expression in 718 gastric cancer cases. Samples with no PPIB or FGFR2 signal were excluded from the analysis

**Fig. 3:**
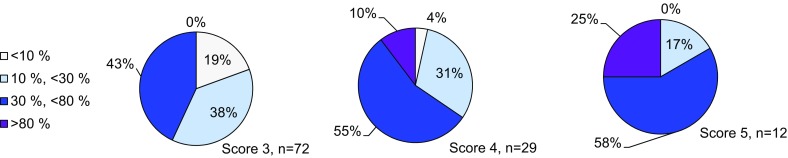
Intratumor heterogeneity of FGFR2 in gastric cancer. The percentage of tumor cells showing FGFR2 staining in either RNAscope (mRNA) or DISH (gene amplification) was determined on tissue slides. Samples with score 3 and 4 RNAscope level were analyzed. Score 5 RNA samples with dense clusters of RNA signal were analyzed separately

**Fig. 4:**
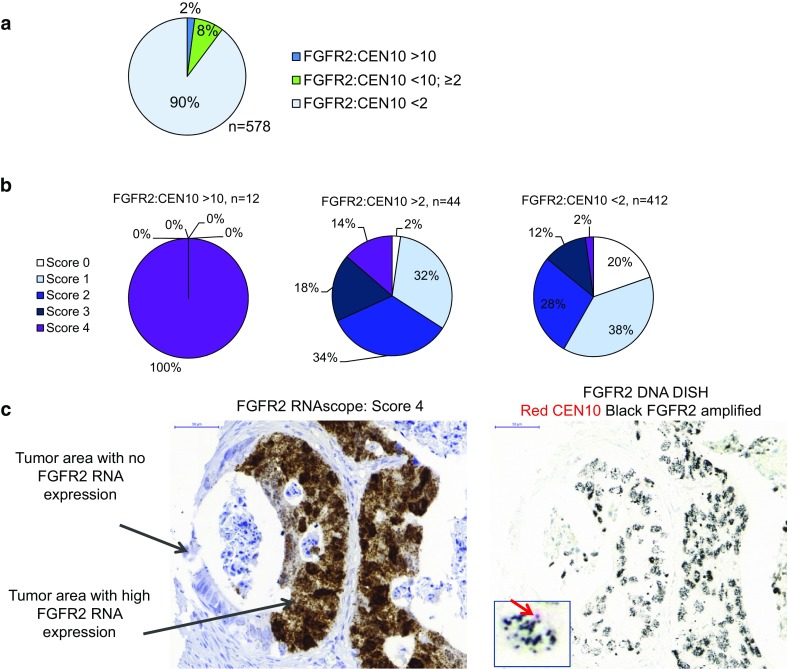
*FGFR2* gene amplification analysis. *FGFR2* gene amplification was determined by dual-color in situ hybridization (DISH) using FGFR2 and CEN10 target probes. High *FGFR2* gene amplification was defined as a FGFR2:CEN10 ratio >10; moderate gene amplification was defined as FGFR2:CEN10 <10 but ≥2. There were no samples with high chromosome 10 polysomy >4 per cell. Data were generated for 578 gastric cancer samples. **a** Prevalence of *FGFR2* gene amplification. **b** Prevalence of FGFR2 mRNA expression by RNAscope in samples with high, moderate, or no *FGFR2* gene amplification **c** FGFR2 RNA scope (*left*) and DISH data (*right*) from the same sample. *Arrows* point to tumor areas with or without FGFR2 mRNA. *Inserted square* shows a high-magnification image of the DISH staining. *Red arrow* points to the CEN10 signal in *red*. FGFR2 signal is *black*

To determine the FGFR2 expression levels in gastric cancer samples and the intratumor heterogeneity of FGFR2, we used the mRNA in situ hybridization technology RNAscope, in which 718 samples of 1036 were successfully analyzed. For 27% of the sample, the RNAscope assay failed (Fig. 1; Supplementary Tables 3, 4). Representative images for FGFR2 and the positive control gene PPIB are shown in (Fig. 2a). Strong FGFR2 expression (score 4) was seen in 4% (29/718) of the samples (Fig. 2b). To investigate if variability in the preservation of mRNA in these specimens affected FGFR2 signals, we analyzed the PPIB control signal and the FGFR2 signal independently (Supplementary Table 4); 259 samples did not give score 0 in either FGFR2 or PPIB. These samples likely did not have sufficient nucleic acid content for the analysis and were excluded from any analysis performed with the RNAscope data. FGFR2 scores 3, 4, and 5 were evenly distributed among the different PPIB scores. The same result was seen for the distribution of the score 3 and 4 PPIB scores among the different FGFR2 scores, suggesting that the detection of the two genes is independent. We therefore used the PPIB score as a quality check. Samples with no signal in either PPIB or FGFR2 were excluded from further analyses. RNAscope is a tissue slide-based method allowing determination of the intratumor heterogeneity of FGFR2 expression. For the samples with moderate and strong FGFR2 expression higher than score 2, we determined the percentage of tumor cells showing FGFR2 mRNA. Two independent cores per sample were analyzed and median values were determined. Following the method described in Nagatsuma et al. (2015), we defined four groups of intratumor heterogeneity: FGFR2 expression in less than 10% of the tumor cells, between 10% and 30%, between 30% and 80%, and larger than 80% (Fig. 3) [22]. FGFR2 mRNA expression was heterogeneous across and within the gastric cancer tissue sample; only 3 of the 718 samples analyzed (0.4%) showed a high (score 3 or 4) FGFR2 mRNA expression level in more than 80% of the tumor cells. We observed a more homogenous FGFR2 expression in tumors with score 4 as compared to score 3. Twelve gastric cancer patients showed very strong FGFR2 mRNA expression with dense clusters of the RNAscope signal visible under a 1× objective. In contrast to the RNAscope scoring algorithm suggested by the kit instructions, we decided to consider these separately. These 12 patients showed an even more homogeneous FGFR2 mRNA expression compared to all score 4 patients (Fig. 3a). An exemplary image of such a case is shown in Fig. 4c. Of note, some tumor cells visible in this image do not show FGFR2 mRNA expression whereas the majority of the cells very strongly express FGFR2. In this case, the FGFR2 RNAscope signal is even visible under a 1× objective.

Representative examples of the DISH analysis are shown in Fig. 2a. The DISH assay was performed on 967 samples. Among the 578 samples successfully analyzed for *FGFR2* gene amplification using DISH 2% (12/578) showed high amplification (FGFR2:CEN10 >10) and 8% (47/578) showed moderate amplification (FGFR2:CEN10 <10; ≥2). The DISH assay failed for 36% of the samples tested (Figs. 1, 4a; Supplementary Tables 3, 4a). We did not observe samples with high chromosome 10 polysomy (>4 per cell). For 468 samples, both RNAscope and DISH data were available. FGFR2 mRNA expression levels were associated with gene amplification; FGFR2 mRNA levels were highest in the highly amplified samples (*n* = 12) and increased in the moderately amplified samples (*n* = 47) (Fig. 4b). Interestingly, all highly amplified samples showed a dense cluster of RNAscope signal, which was also visible under a 1× objective. The identical cells in the tissue showed high *FGFR2* gene amplification (FGFR2:CEN10 >10) and dense clusters of FGFR2 RNAscope signal (Fig. 4c). These data indicate that very high FGFR2 mRNA expression was associated with high *FGFR2* gene amplification in gastric cancer. These samples also showed a more homogeneous expression within the tumor (Fig. 3). Based on these findings, we suggest to expand the scoring algorithm of the RNAscope kit and add a score 5 category with dense clusters of RNAscope signal visible under a 1× objective.

**Fig. 5:**
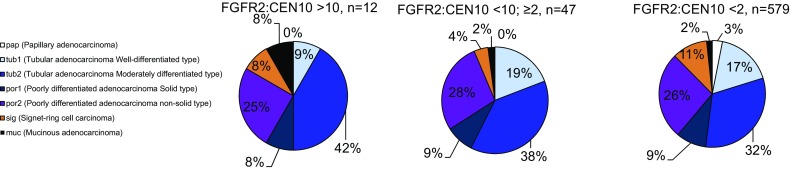
Gastric cancer histology versus *FGFR2* gene amplification. Histological subtypes were determined following the Japanese classification of gastric carcinoma. Prevalence of histological subtypes in samples with high, moderate, or no *FGFR2* gene amplification is plotted

We investigated the correlation of *FGFR2* gene amplification and histological subtype (Fig. 5a). Samples with high *FGFR2* gene amplification were both of intestinal subtype (papillary or tubular adenocarcinoma) and diffuse type (poorly differentiated adenocarcinoma and signet-ring cell carcinoma). There was no difference in the prevalence of histological subtypes between highly *FGFR2* amplified or non-amplified gastric cancer samples. Overall, there was no statistical significant relationship between histological subtype and *FGFR2* gene amplification in this study (Fig. 5a).

**Fig. 6:**
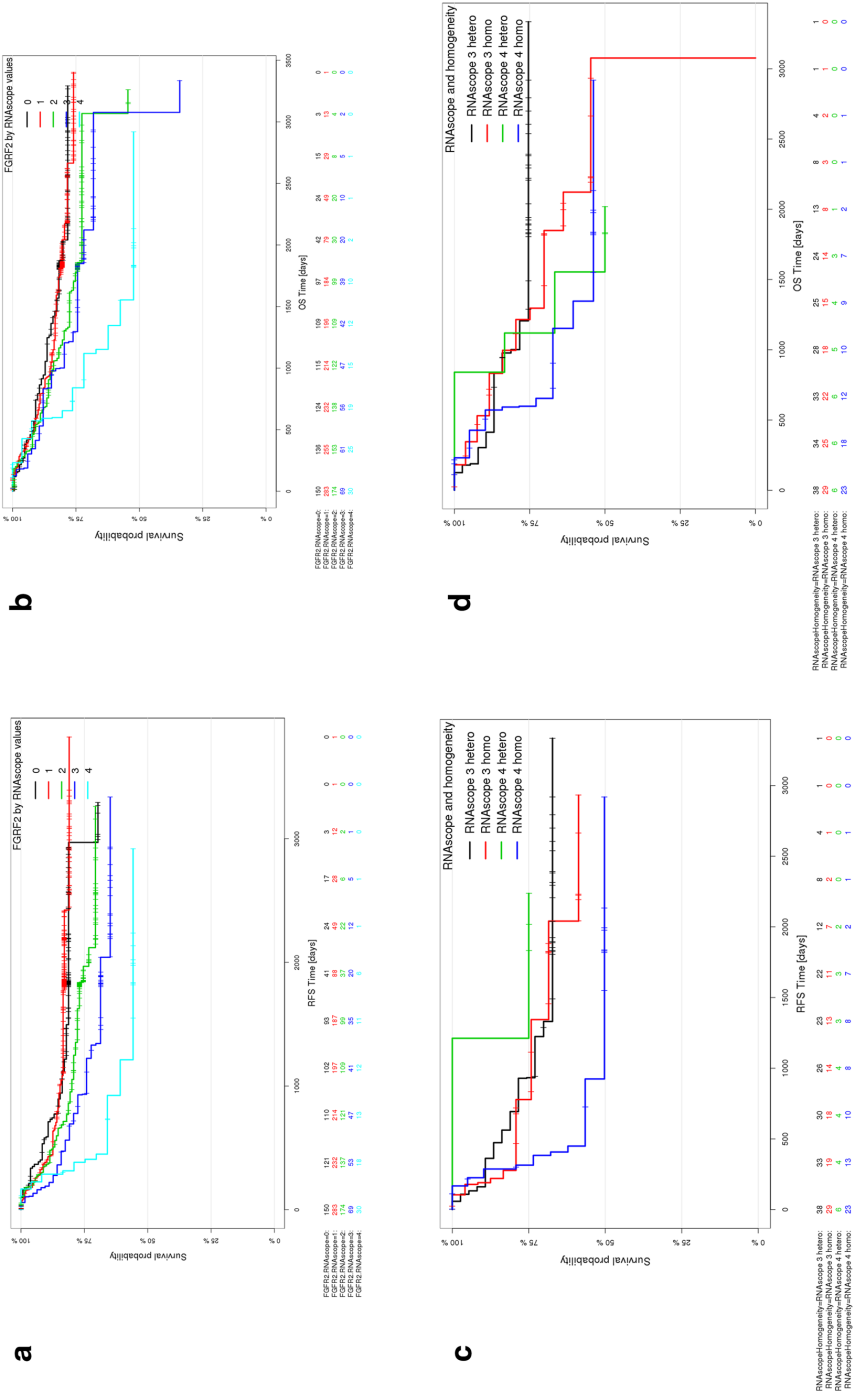
Patient outcomes according to FGFR2 mRNA level. **a** Kaplan–Meier plot for recurrence-free survival (RFS) for the FGFR2 RNA levels determined by RNAscope (0–4). **b** Kaplan–Meier plot for overall survival (OS) for the FGFR2 RNA levels determined by RNAscope (0–4). **c** Kaplan–Meier plot for RFS in the FGFR2 RNAscope groups score 3 and 4 with heterogeneous (≤30% of tumor cells show expression) and homogenous expression (≥30%). **d** Kaplan–Meier plot for OS in the FGFR2 RNAscope groups score 3 and 4 with heterogeneous and homogenous expression

We tested the correlation of patient outcome with FGFR2 mRNA expression as determined by RNAscope. A univariate Cox proportional hazards (Cox PH) and Kaplan–Meier analysis suggested that FGFR2 mRNA expression levels by RNAscope were associated with relapse-free survival [RFS: HR = 1.308; 95% CI = (1.122–1.525); *p* = 0.0006] (Fig. 6a). Gastric cancer patients who had an FGFR2 mRNA expression score of 4 had a shorter RFS compared to patients with a sample score of 0–3. However, after adjusting the Cox PH model for clinical covariates (UICC stage and macroscopic classification were the only ones selected by the AIC algorithm), the association between FGFR2 mRNA expression levels and RFS was no longer significant [HR = 1.133; 95% CI = (0.9740–1.317); *p* = 0.11]. The clinical significance did not improve when analyzing tumor stage I/II/III/IV separately (Supplementary Figure 1). An overview of FGFR2 status in various clinicopathological subgroups is shown in Supplementary Tables 5 and 6.

In a univariate Cox PH and Kaplan–Meier analysis, FGFR2 mRNA expression levels were associated with overall survival [OS: HR = 1.256, 95% CI = (1.085–1.454); *p* = 0.002] (Fig. 6b). When adjusting the Cox PH model for clinical covariates, association of FGFR2 mRNA expression level with OS was generally discarded from the model through AIC-based forward-backward selection. The algorithm selected gender, age, UICC stage, and macroscopic classification as the only variables with prognostic relevance for OS. Similar to the RFS analysis, separate correlation by tumor stage did not result in clinical significance. We were interested whether intratumor heterogeneity of FGFR2 mRNA expression is associated with differences in RFS. Figure 6c shows a Kaplan–Meier plot for RFS in the FGFR2 RNAscope groups of score 3 and 4 with heterogeneous (≤30% of tumor cells show expression) and homogeneous expression (≥30%). Even though patients with homogeneous score 4 FGFR2 mRNA expression had the shortest RFS, the differences between the groups are not significant. Intratumor heterogeneity of FGFR2 mRNA expression also did not appear to be associated with OS (Fig. 6d).

**Fig. 7:**
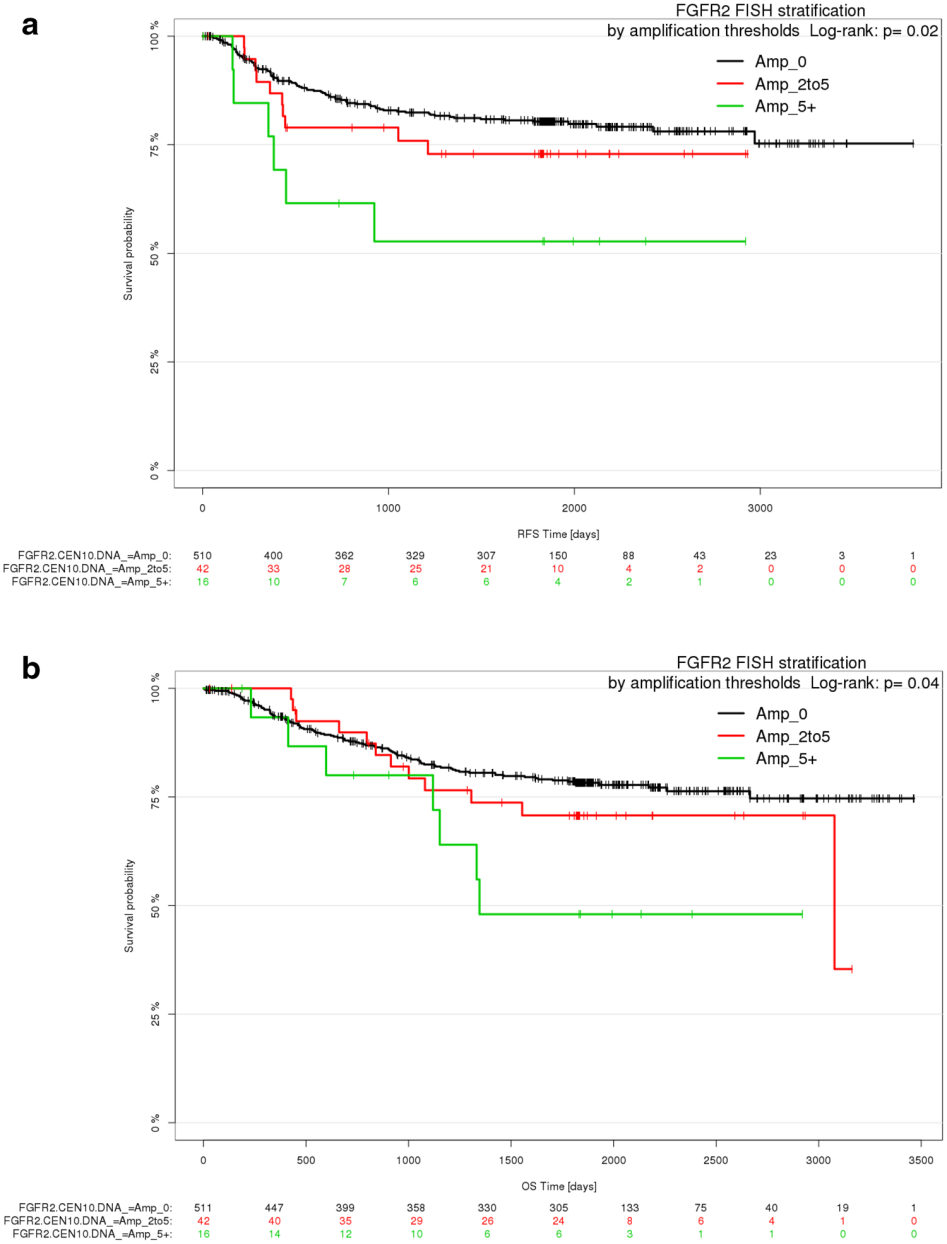
Patient outcomes according to *FGFR2* gene amplification. **a** Kaplan–Meier plot for RFS for the *FGFR2* gene amplification levels determined by DISH. **b** Kaplan–Meier plot for OS for the *FGFR2* gene amplification levels determined by DISH

Next, we investigated the correlation of *FGFR2* gene amplification and patient outcome data. Univariate Cox PH and Kaplan–Meier analysis suggested that patients with tumors that had *FGFR2* gene amplification had shorter RFS than patients with nonamplified tumors (HR = 1.016; 95% CI = 1.001–1.031; *p* = 0.03) (Fig. 7a). After adjusting the Cox PH model for clinical covariates, *FGFR2* gene amplification status was not significantly associated with RFS and was discarded from the model based on AIC-based forward-backward selection. Neither in univariate nor in multivariate Cox PH and Kaplan–Meier analyses was *FGFR2* gene amplification significantly associated with OS (Fig. 7b).

## Discussion

FGFR2 has been proposed as a target for the treatment of gastric cancer [24]. Several FGFR2-targeted agents have recently been described, including anti-FGFR2 antibodies and FGFR2-ADCs and dual FGFR2/FGFR4 targeting ADCs, as well as small molecular inhibitors with activity against FGFR2 [25–34]. Clinical studies selecting FGFR2-positive gastric cancer patients based on *FGFR2* gene amplification with different methods are currently ongoing; however, no FGFR2-targeted agent along with a biomarker assay has been approved yet. FGFR2 mRNA expression rather than *FGFR2* gene amplification has been discussed as a biomarker to select patients for FGFR2-targeted therapies in gastric cancer [35]. We wanted to investigate both *FGFR2* gene amplification and mRNA expression as biomarkers in gastric cancer. The gold standard to detect *FGFR2* gene amplification is FISH. However, the method is expensive and requires fluorescence microscopy, limiting its use in clinical practice. Polymerase chain reaction (PCR)-based methods are cheaper but lack spatial resolution; the latter might be problematic considering that gastric cancer has been shown to have a very high rate of intratumor heterogeneity [36]. We therefore explored a novel mRNA in situ technique to detect FGFR2 mRNA and the intratumor heterogeneity. We compared the results with *FGFR2* gene amplification determined with another in situ technique, dual-color in situ hybridization (DISH), which does not require fluorescence microscopy and is more convenient to use compared to FISH. Very recently, Han et al. performed a study exploring FGFR2 mRNA expression by in situ techniques and FGFR2 protein expression by IHC in 362 surgically resected gastric cancer tissues and 135 matched metastatic lymph nodes from Korea. *FGFR2* gene amplification was determined by FISH in 188 of the samples [18]. Given the high medical need in gastric cancer and the high interest in FGFR2 as a target for gastric cancer, we set out to investigate FGFR2 in a larger cohort of Japanese gastric cancer patients.

Not all the available samples were analyzed by DISH; 1036 samples were analyzed by RNAscope and only 967 of those were also analyzed by DISH. Some samples could not be analyzed because the core in the TMA was missing or the sample did not have sufficient tumor content. The percentage of successful analysis of samples was acceptable, with 69% for RNAscope and 60% for DISH (Supplementary Table 3). For 27% of the tested samples the RNAScope assay failed; 36% of the assayed DISH samples did not give a result. The samples analyzed in this study were fixed in unbuffered formalin and stored for more than 10 years, explaining the rather high failure rate. Both in situ techniques detect nucleic acids, and factors preserving or degenerating nucleic acids will affect both methods in a similar way. In line with that expectation, 68% of the samples tested either failed both assays or were successfully analyzed with both methods (Supplementary Table 3). There was no major difference in failure rate between the two methods. The success rate is slightly higher for RNAscope. We cannot exclude the possibility that degradation of nucleic acids in some samples biased the analysis. FGFR2 mRNA was successfully determined in 718 patients and *FGFR2* gene amplification in 578 patients. We were able to generate both *FGFR2* gene amplification and mRNA expression for 468 patients. We detected a high level of *FGFR2* gene amplification (FGFR2:CEN10 >10) in 2% (12/578) of the samples, and high FGFR2 mRNA expression score 4 was detected in 4% (29/718) of samples. Moderate *FGFR2* gene amplification (FGFR2:CEN10 >2, <10) was detected in 8% (47/578). Previous studies have reported *FGFR2* gene amplification in 3.6–9% of gastric cancer cases [17, 35, 37–43], which is in line with our findings. Han et al. reported 2.7% *FGFR2* gene amplification and 5.8% FGFR2 mRNA overexpression, which is very similar to the 2% of cases with high *FGFR2* gene amplification and 4% mRNA score 4 patients we detected in a large cohort of Japanese gastric cancer patients [18].

We assessed intratumor heterogeneity using TMAs. The small size of TMA cores as compared to whole-tissue sections is limiting this analysis. As recommended by previous reports, we sought to overcome this limitation by obtaining two cores from different areas to evaluate the tissue heterogeneity, with one sample collected from the intramural area and one from the invasive front area. The statistical power given by the large sample size is thought to compensate for the small size of the TMA cores [44, 45]. In our study, tumor areas with high *FGFR2* gene amplification also showed high levels of FGFR2 mRNA expression. All samples with dense clusters of RNAscope signal, visible under a 1× objective, showed a high level of *FGFR2* gene amplification with a FGFR2:CEN10 ration >10. Surprisingly, these samples also showed a more homogeneous FGFR2 expression within the tumor sample, suggesting that *FGFR2* gene amplification might have been an early event in the development of these gastric cancer cases. The scoring algorithm described in the RNAscope kit instructions does not allow separating this potentially biologically distinct subgroup from regular score 4 cases. We therefore suggest adding a score 5 category for samples with dense clusters of RNAscope signal visible under a 1× objective. There were only 12 score 5 samples with high *FGFR2* gene amplification in our study. Further studies may help to explore if this subgroup is indeed biologically distinct from other gastric cancer patients.

Several studies have described the association of *FGFR2* gene amplification with histological subtypes [46, 47]. The gastric cancer samples used in this study have been histologically classified according to the Japanese Classification of Gastric Cancer [23]. We did not find a statistical significant relationship between histological subtype and *FGFR2* gene amplification in this study (Fig. 5a).

Preclinical studies in gastric cancer cell lines such as SNU-16 have shown impressive efficacy of anti-FGFR2 targeted agents [25, 43]. However, in contrast to clonal cell lines, clinical gastric cancer samples show a high level of intratumor heterogeneity [38]. Gastric cancer cases with focal gene amplification of *HER2* and *FGFR2* in different parts of the tumor tissue have been reported [39, 41, 48, 49], which might lead to insufficient clinical activity or resistance of targeted therapies in gastric cancer. In line with previous reports we find here FGFR2 mRNA expression and gene amplification to be heterogeneous in gastric cancer tissue [19, 38, 41]. Our report is the largest study so far to investigate the heterogeneity of these FGFR2 biomarkers. We found only 0.4% of the investigated gastric cancer cases with high and homogeneous FGFR2 expression, limiting the value of FGFR2 as a target in gastric cancer. However, samples with high FGFR2 mRNA expression intensity also showed a more homogeneous expression pattern as compared to samples with a moderate FGFR2 mRNA expression intensity. We previously reported that high levels of tyrosine kinase receptor (RTK) gene amplification of EGFR, HER2, FGFR2, or MET had protein overexpression and rarely showed other coexisting gene alterations using the next-generation sequencing (NGS) method for gastric cancer [50]. This finding might indicate that alteration in FGFR2 expression occurred early in these cases and may be an oncogenic driver. For these rare cases with high and homogeneous FGFR2 expression, an anti-FGFR2 target agents might be a treatment option in monotherapy. The SHINE trial, which studied the small molecular FGFR1/2/3 inhibitor AZD4547 versus paclitaxel in advanced gastric cancer with *FGFR2* polysomy or gene amplification, did not show any statistically significant difference in PFS in favor of the AZD4547 arm compared with paclitaxel. The authors report intratumor heterogeneity of *FGFR2* gene amplification in the treated patients, suggesting this might have limited the activity of AZD4547 in this trial [49]. One can speculate that focusing on these rare gastric cancer patients with high homogenous FGFR2 expression may have resulted in higher efficacy for AZD4547.

In our study, gastric cancer patients with high FGFR2 mRNA expression with a score of 4 had shorter RFS compared with patients with tumors that had a score of 0–3. Cox PH-based analyses showed a modest association of FGFR2 mRNA expression with both RFS and OS. After adjusting the models for major clinical covariates such as staging, this association was not statistically significant. *FGFR2* gene amplification was also associated with RFS but not with OS in an invariant analysis, and does not appear to be an independent prognostic predictor of RFS in gastric cancer. In comparison, FGFR2 mRNA levels determined by RNAscope showed a stronger association with clinical outcome as compared to *FGFR2* gene amplification. We show here that RNAscope is a valuable tool to confirm target expression in tumor samples. Recently, the suitability of RNAscope for detection of HER2 mRNA in breast cancer was demonstrated, and the sensitivity was shown to be higher than that of FISH for gene amplification. Based on mRNA analysis, an additional 7% of breast cancer patients may be eligible for Herceptin therapy [51]. HER2 detection by RNAscope correlates well with immunohistochemistry and DNA-FISH [52]. HER2 expression could also be detected reliably by RNAscope in gastric cancer [53].

Our study is the largest so far to report FGFR2 detection by RNAscope compared to gene amplification by DISH in gastric cancer. Overall, our study demonstrated that RNAscope and DISH are suitable methods to evaluate FGFR2 status in gastric cancer FFPE tissue slides, allowing evaluation of the intratumor heterogeneity of these FGFR2 biomarkers.

Our study showed that high *FGFR2* gene amplification and high FGFR2 mRNA expression were associated with a more homogeneous presence of the markers. There was a trend for a shorter RFS for patients with high and homogenous FGFR2 expression, suggesting this might be a target population for FGFR2-targeted therapies.

In conclusion, mRNA and DNA in situ methods are suitable to determine FGFR2 mRNA expression and *FGFR2* gene amplification and the respective intratumor heterogeneity. We propose that an analysis of FGFR2 mRNA and DNA status and the respective intratumor heterogeneity in clinical studies with FGFR2-targeted agents will be necessary to identify the right patient selection strategy for FGFR2-directed therapies.

## Electronic supplementary material

Below is the link to the electronic supplementary material.

Supplementary material 1 (PPTX 585 kb)

Supplementary material 2 (XLSX 12 kb)

Supplementary material 3 (XLSX 11 kb)

Supplementary material 4 (XLSX 12 kb)
