# Supplementary material for: In situ analysis of FGFR2 mRNA and comparison with FGFR2 gene copy number by dual-color in situ hybridization in a large cohort of gastric cancer patients
Source: Gastric Cancer. 2017 Aug 29;21(3):401–12. doi: 10.1007/s10120-017-0758-x (PMC5906494; doi:10.1007/s10120-017-0758-x)
Supplement: Supplementary file 1 — Supplementary material 1 (PPTX 585 kb) [file 10120_2017_758_MOESM1_ESM.pptx]

## Slide 1
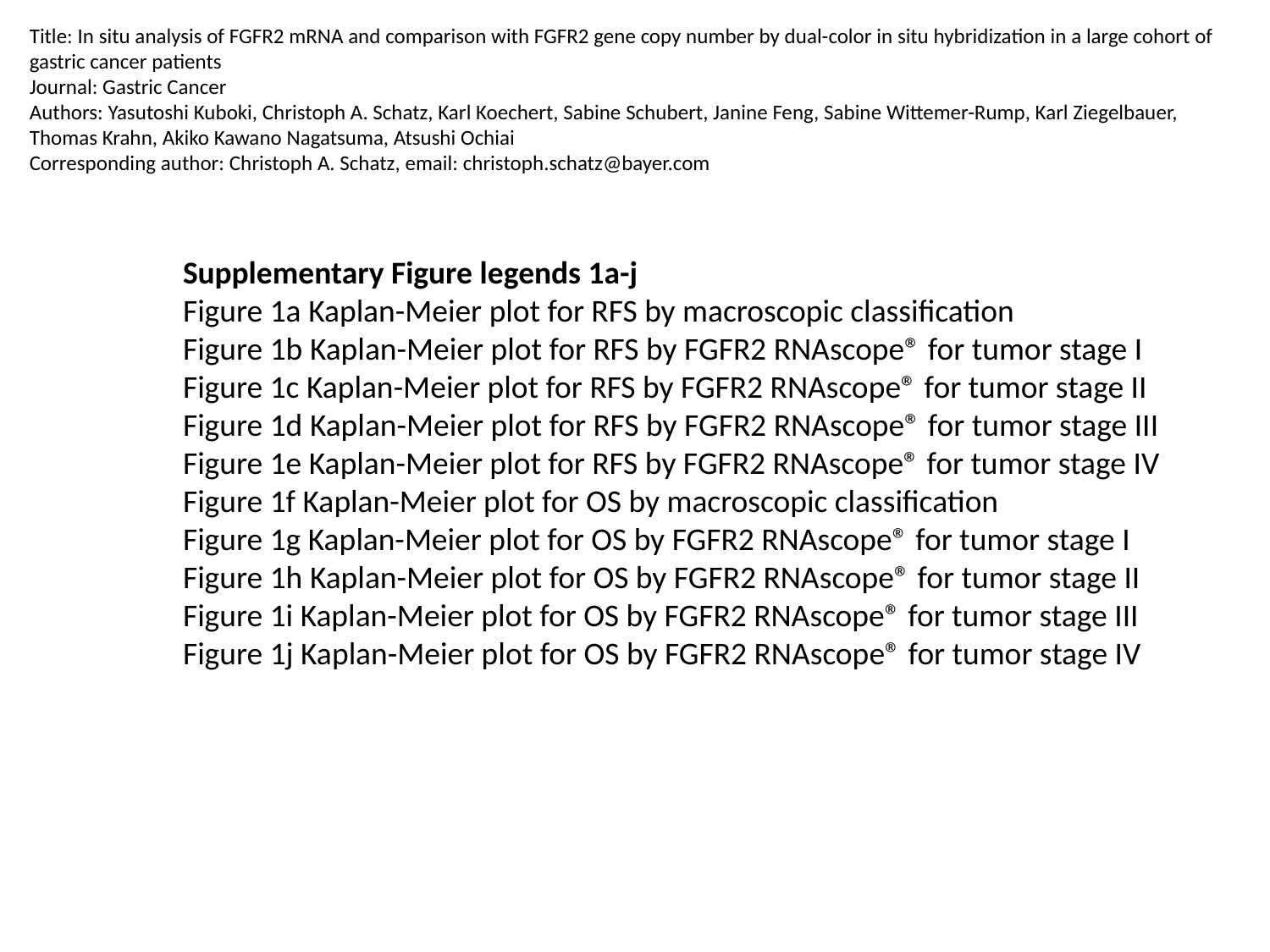

Title: In situ analysis of FGFR2 mRNA and comparison with FGFR2 gene copy number by dual-color in situ hybridization in a large cohort of gastric cancer patients
Journal: Gastric Cancer
Authors: Yasutoshi Kuboki, Christoph A. Schatz, Karl Koechert, Sabine Schubert, Janine Feng, Sabine Wittemer-Rump, Karl Ziegelbauer, Thomas Krahn, Akiko Kawano Nagatsuma, Atsushi Ochiai
Corresponding author: Christoph A. Schatz, email: christoph.schatz@bayer.com
Supplementary Figure legends 1a-j
Figure 1a Kaplan-Meier plot for RFS by macroscopic classification
Figure 1b Kaplan-Meier plot for RFS by FGFR2 RNAscope® for tumor stage I
Figure 1c Kaplan-Meier plot for RFS by FGFR2 RNAscope® for tumor stage II
Figure 1d Kaplan-Meier plot for RFS by FGFR2 RNAscope® for tumor stage III
Figure 1e Kaplan-Meier plot for RFS by FGFR2 RNAscope® for tumor stage IV
Figure 1f Kaplan-Meier plot for OS by macroscopic classification
Figure 1g Kaplan-Meier plot for OS by FGFR2 RNAscope® for tumor stage I
Figure 1h Kaplan-Meier plot for OS by FGFR2 RNAscope® for tumor stage II
Figure 1i Kaplan-Meier plot for OS by FGFR2 RNAscope® for tumor stage III
Figure 1j Kaplan-Meier plot for OS by FGFR2 RNAscope® for tumor stage IV

## Slide 2
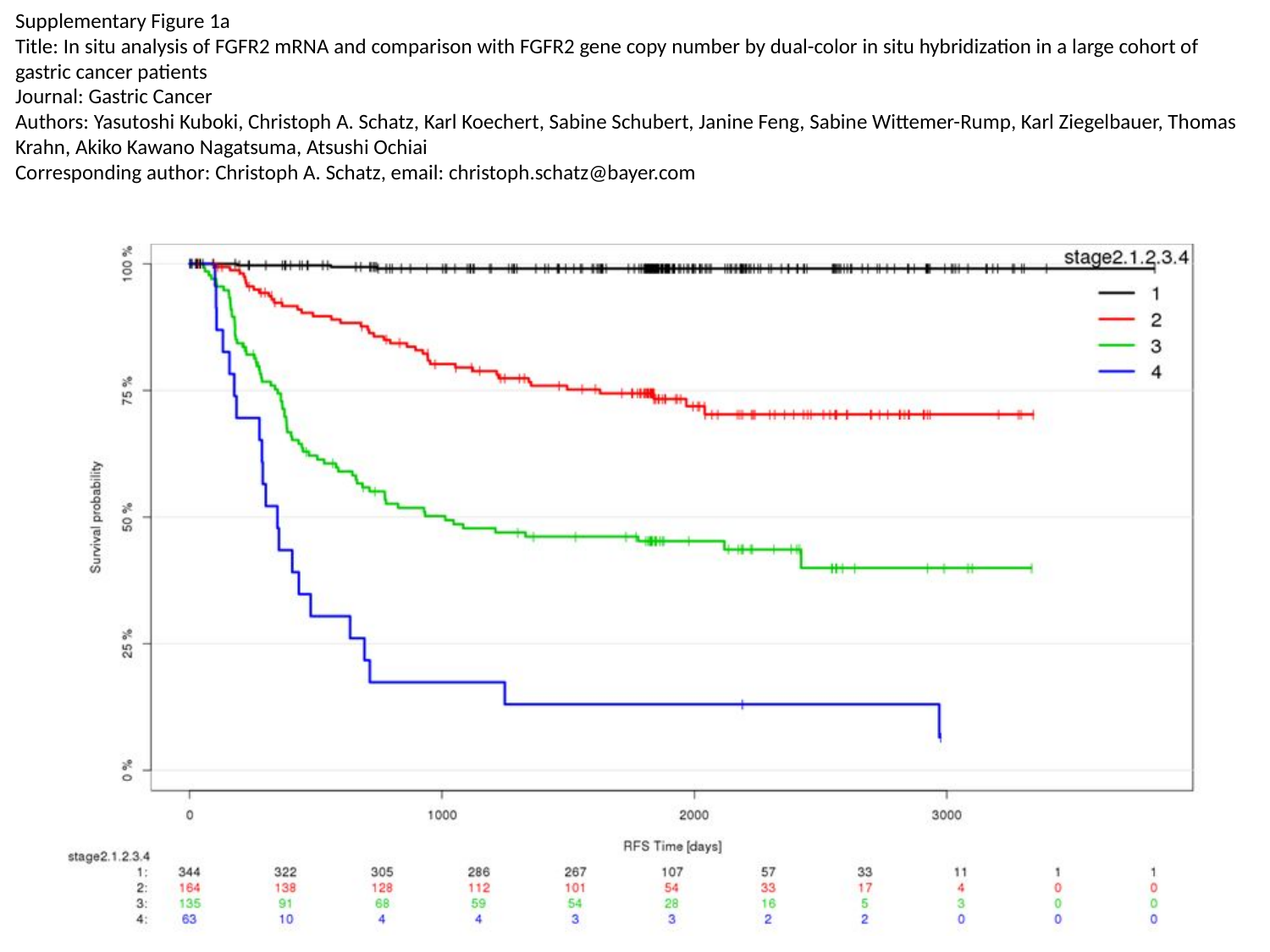

Supplementary Figure 1a
Title: In situ analysis of FGFR2 mRNA and comparison with FGFR2 gene copy number by dual-color in situ hybridization in a large cohort of gastric cancer patients
Journal: Gastric Cancer
Authors: Yasutoshi Kuboki, Christoph A. Schatz, Karl Koechert, Sabine Schubert, Janine Feng, Sabine Wittemer-Rump, Karl Ziegelbauer, Thomas Krahn, Akiko Kawano Nagatsuma, Atsushi Ochiai
Corresponding author: Christoph A. Schatz, email: christoph.schatz@bayer.com

## Slide 3
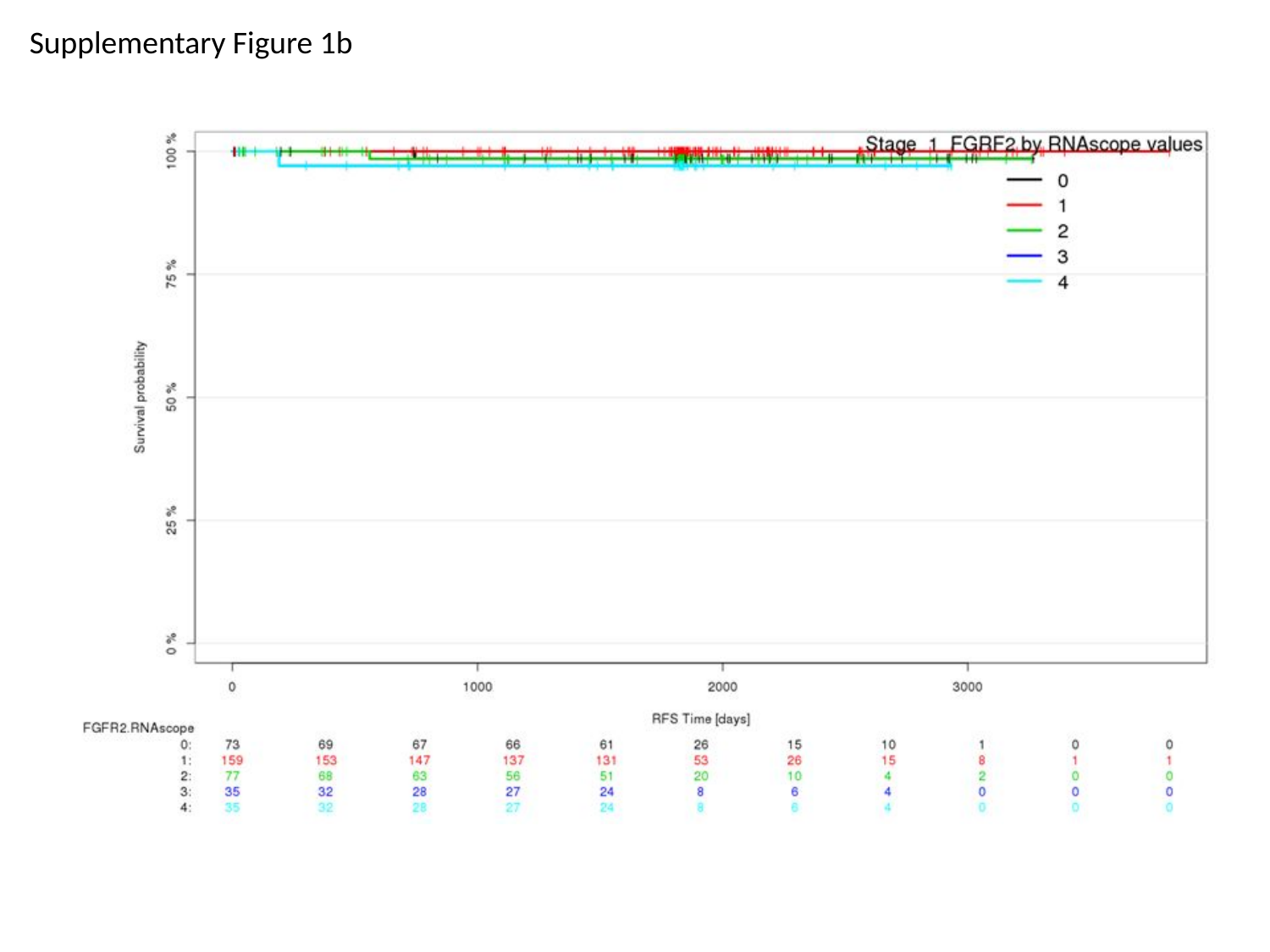

Supplementary Figure 1b

## Slide 4
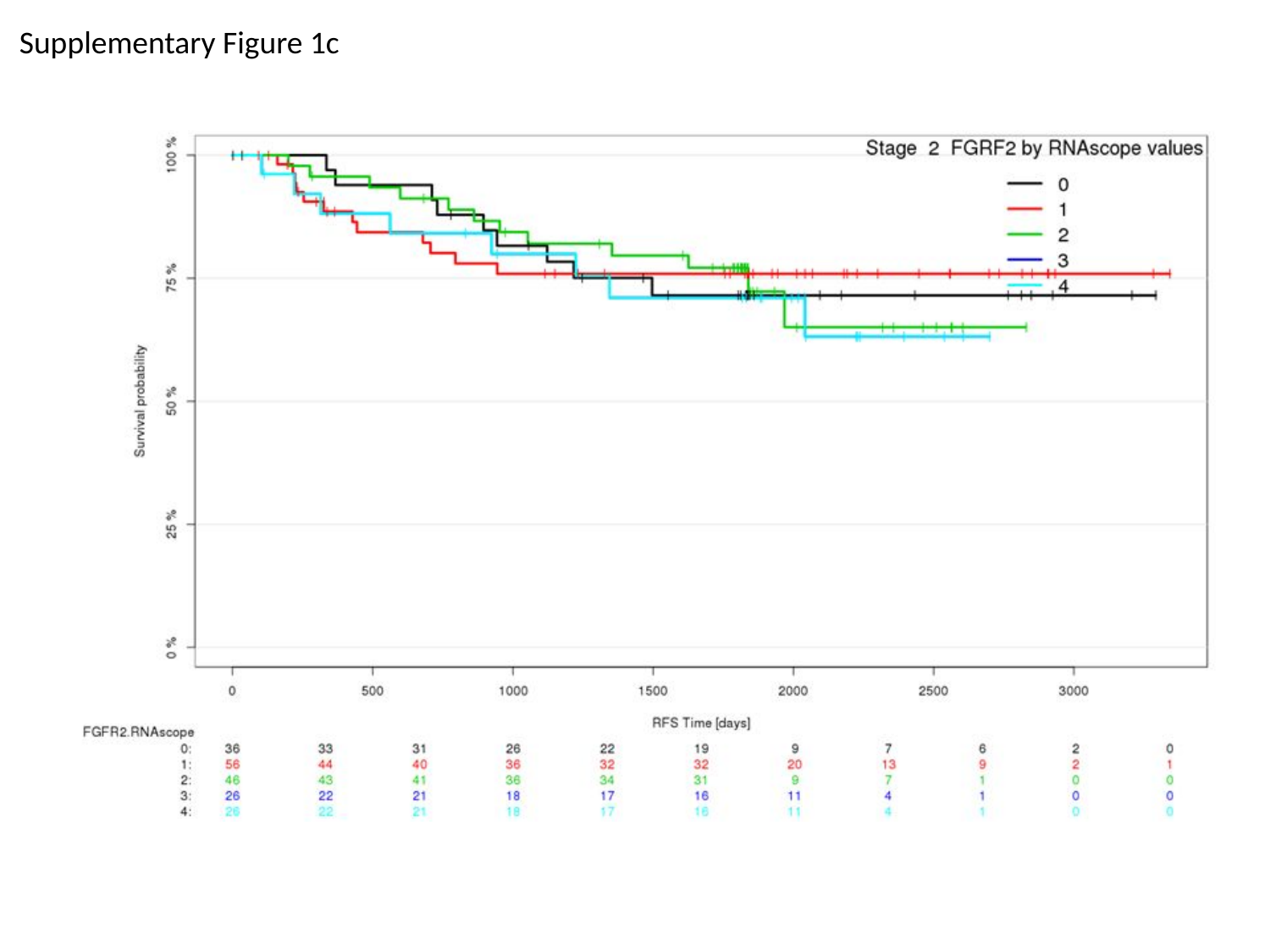

Supplementary Figure 1c

## Slide 5
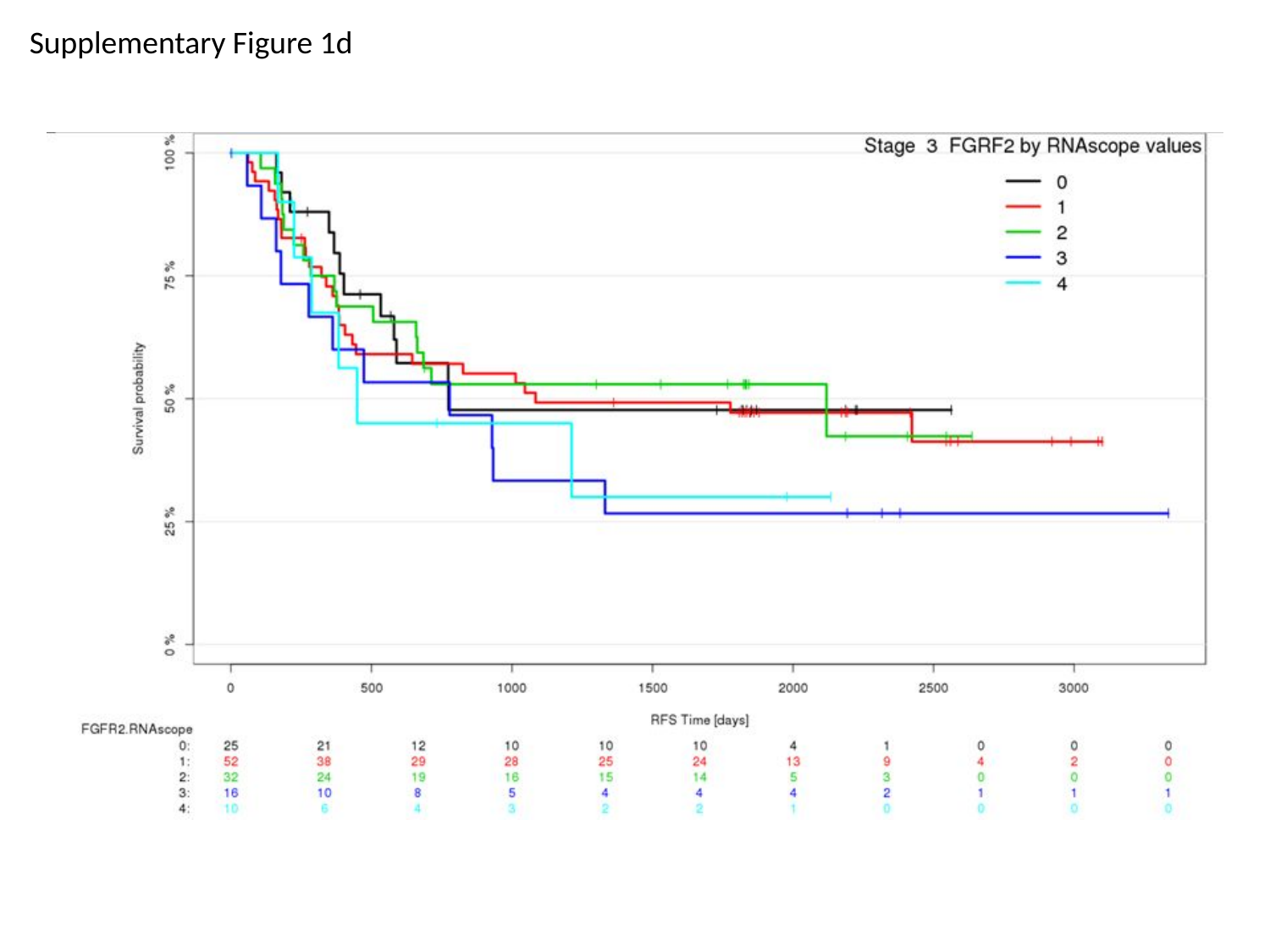

Supplementary Figure 1d

## Slide 6
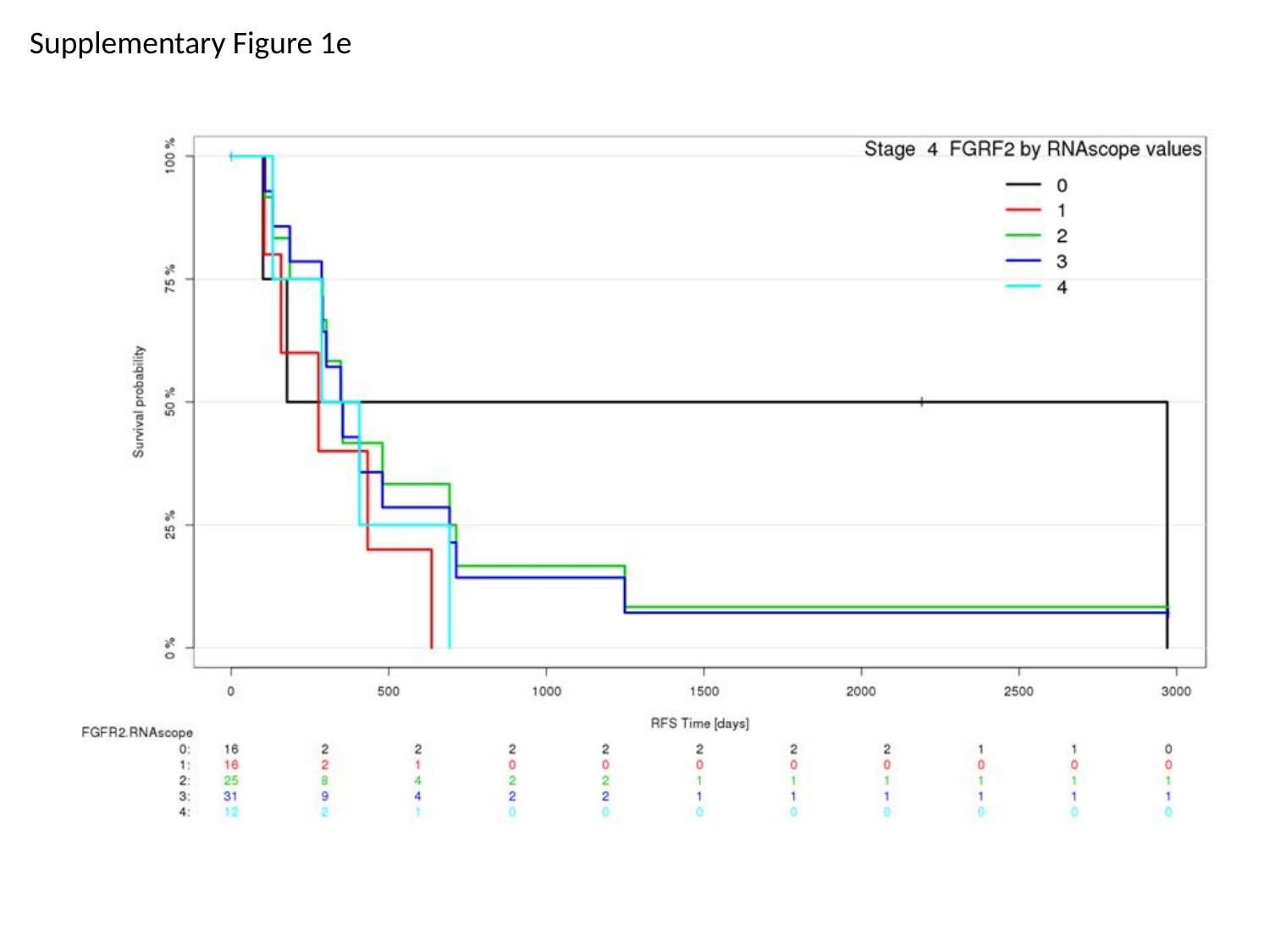

Supplementary Figure 1e

## Slide 7
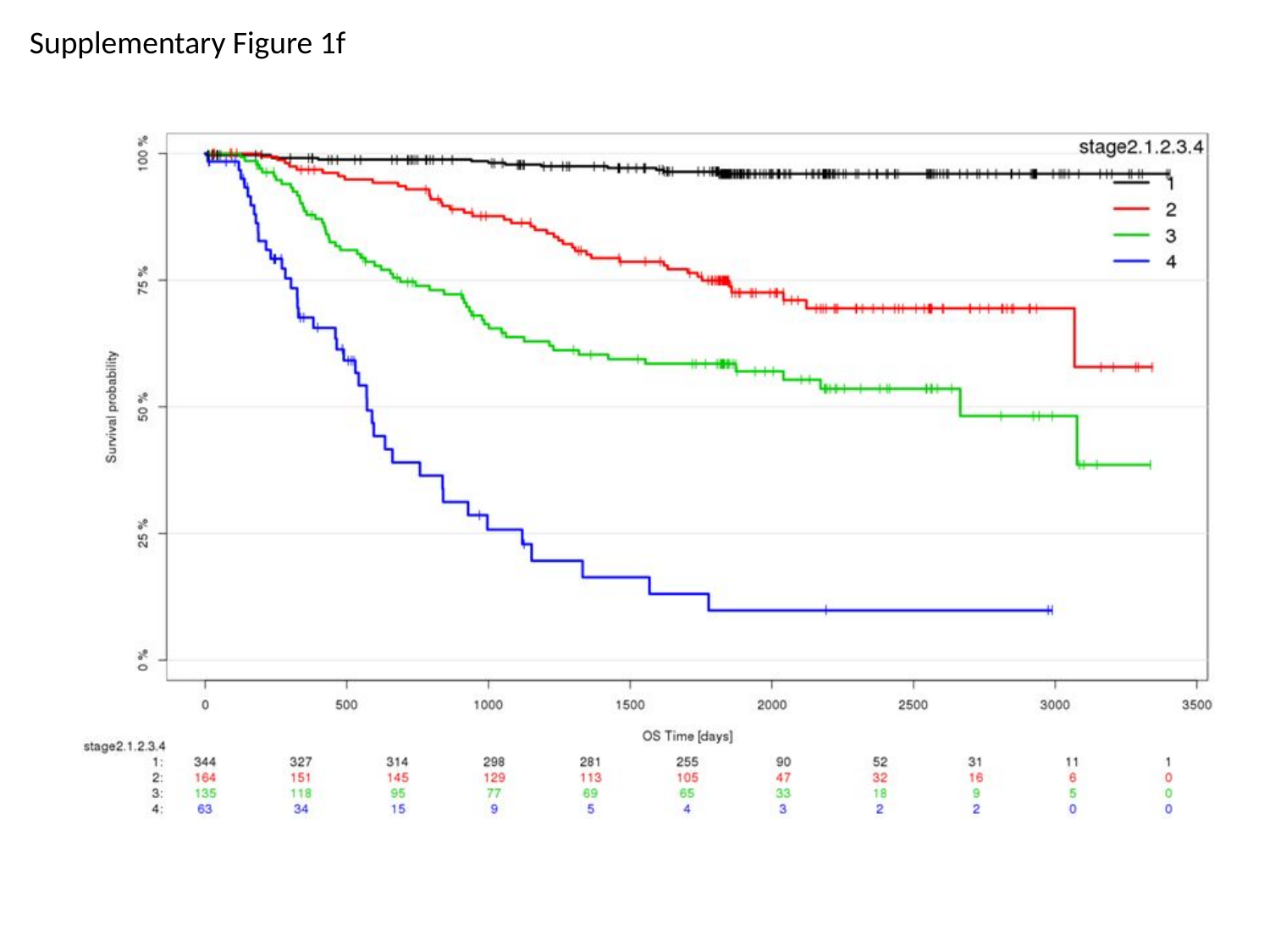

Supplementary Figure 1f

## Slide 8
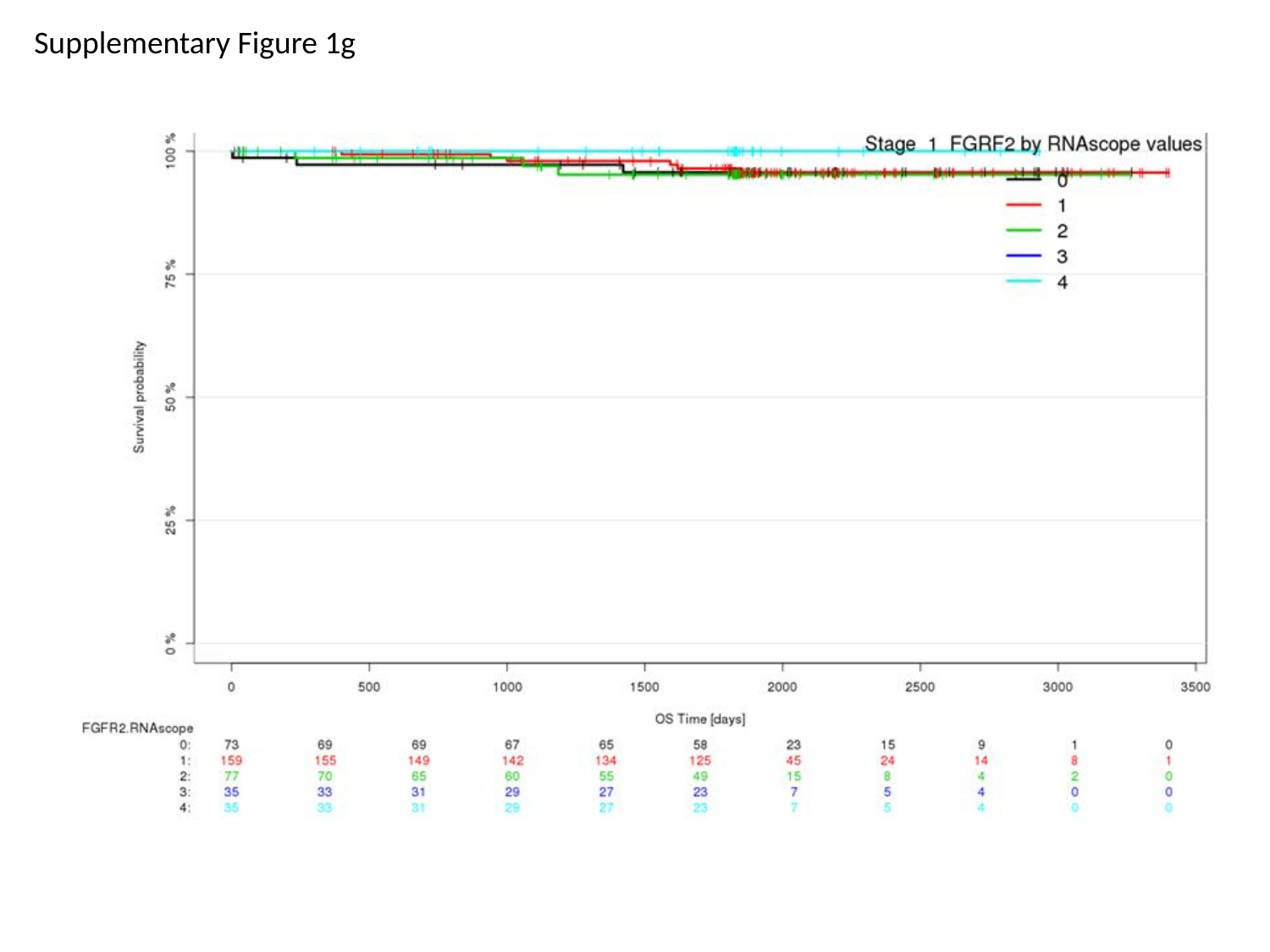

Supplementary Figure 1g

## Slide 9
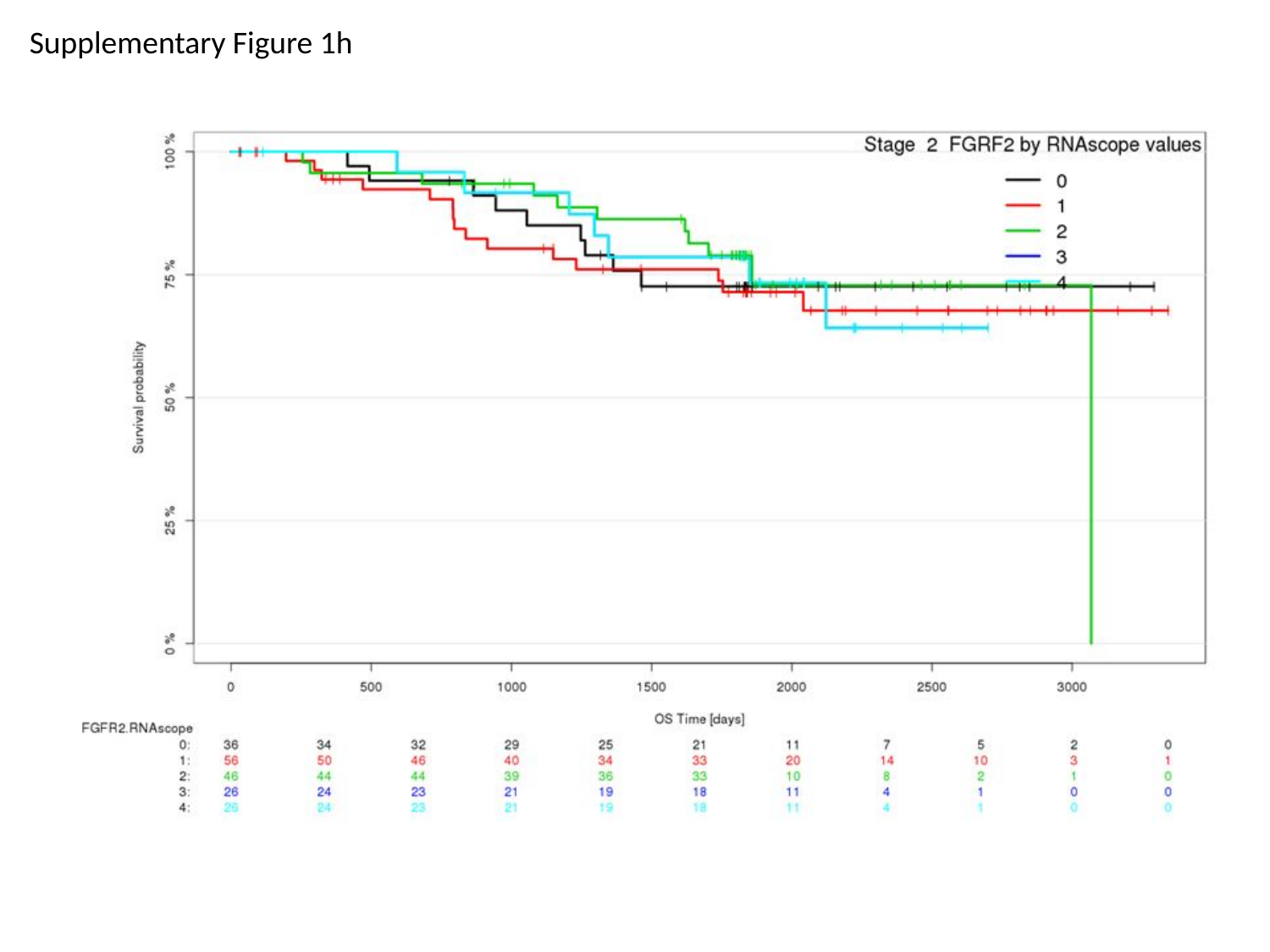

Supplementary Figure 1h

## Slide 10
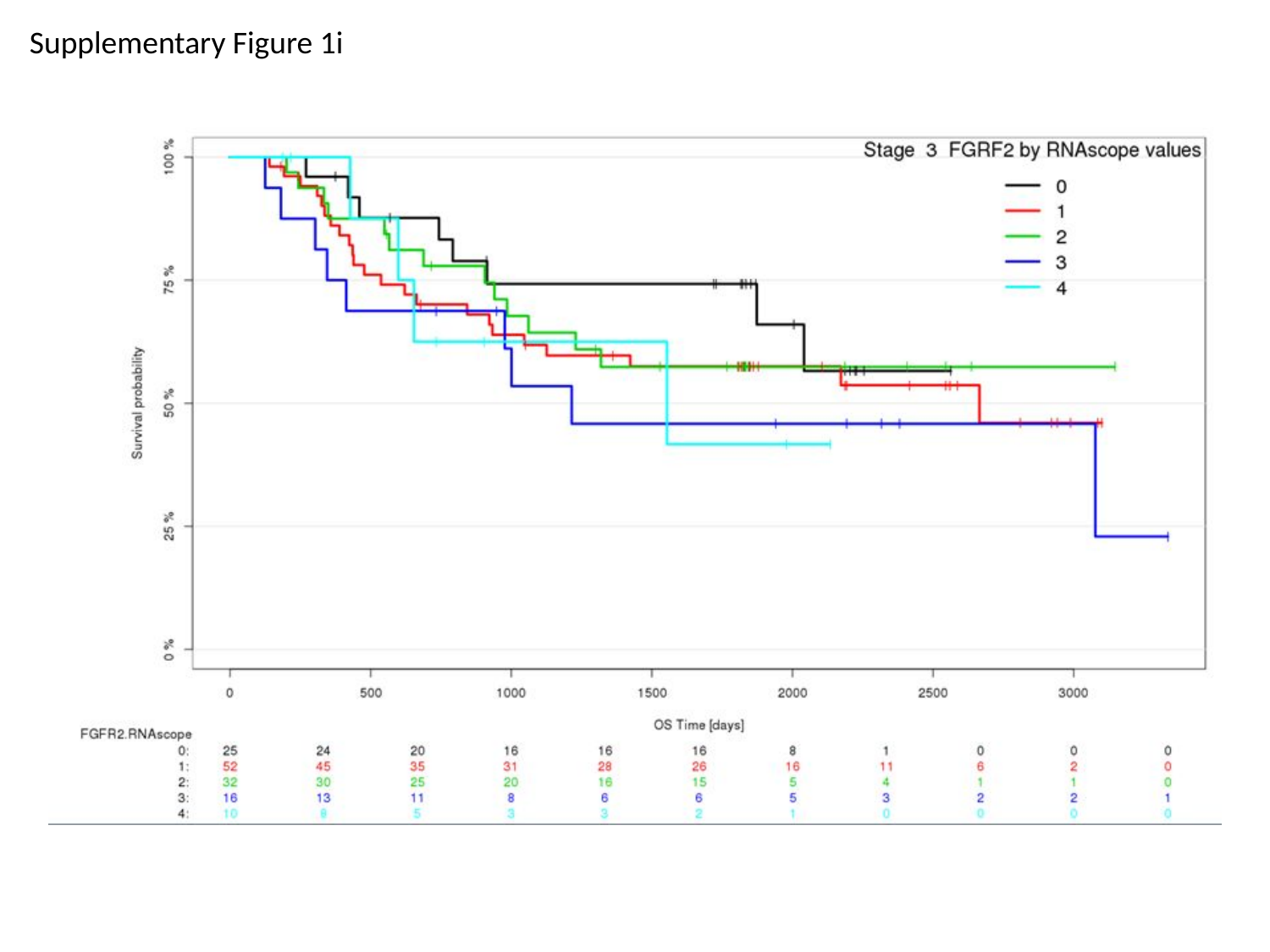

Supplementary Figure 1i

## Slide 11
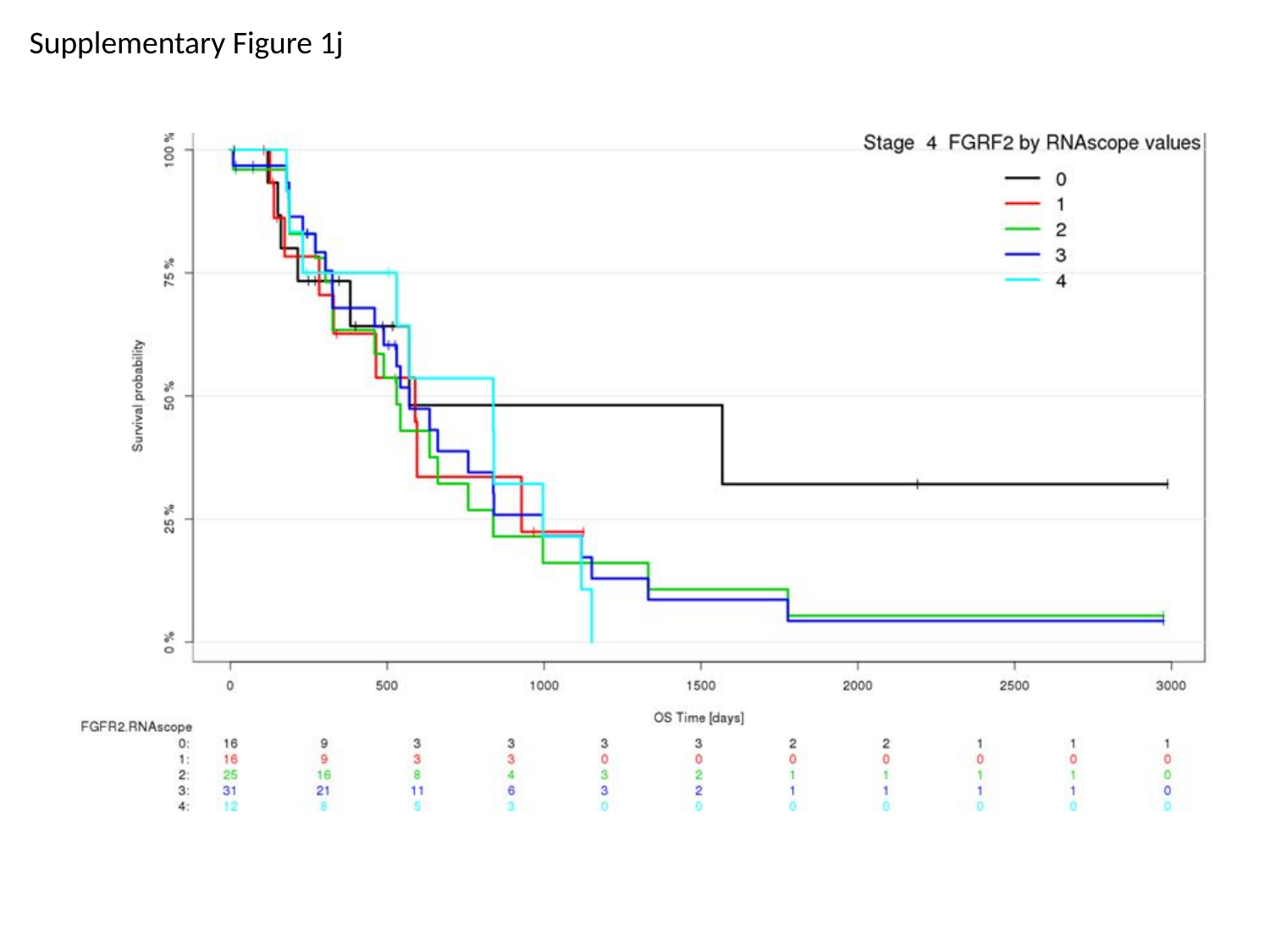

Supplementary Figure 1j
